# Fibrotic Disease of the Skin and Lung: Shared Pathways, Environmental Drivers, and Therapeutic Opportunities in a Changing Climate

**DOI:** 10.3390/ijms26178394

**Published:** 2025-08-29

**Authors:** Katerina Grafanaki, Alexandros Maniatis, Vasilina Sotiropoulou, Efstathia Pasmatzi, Argyris Tzouvelekis

**Affiliations:** 1 Department of Dermatology-Venereology, School of Medicine, University of Patras, 26504 Patras, Greece; 2 Department of Biochemistry, School of Medicine, University of Patras, 26504 Patras, Greece; aalexmaniatis1999@gmail.com; 3 Department of Respiratory Medicine, School of Medicine, University Hospital of Patras, 26504 Patras, Greeceargyris.tzouvelekis@gmail.com (A.T.)

**Keywords:** skin fibrosis, pulmonary fibrosis, fibroinflammatory remodeling, TGF-b signaling, IL-4/IL-13 axis, exposome, epigenetic regulation, microRNA, climate change, precision antifibrotic therapy, drug repurposing

## Abstract

Fibrotic diseases of the skin and lung, such as systemic sclerosis, hypertrophic scars, keloids, and pulmonary fibrosis, share core molecular mechanisms despite their distinct anatomical settings. Central to their pathogenesis are persistent fibroblast activation, immune dysregulation, ECM remodeling, and failure of resolution pathways, all modulated by an ever-changing environment and epigenetic regulation. Increasing evidence reveals that chronic injury from air pollution, ultraviolet radiation, climate stressors, and occupational hazards accelerates fibroinflammatory remodeling across these barrier organs. Moreover, shared signaling networks, including TGF-β, IL-4/IL-13, Wnt/β-catenin, and epigenetic regulators like miR-21 and miR-29, suggest convergent fibrotic programs may be subject to cross-organ therapeutic targeting. This review integrates recent insights into the exposome’s role in driving fibrosis, highlights novel RNA- and epigenetic-based interventions, and evaluates the repurposing of antifibrotic agents approved for pulmonary disease within dermatologic contexts. We emphasize the emerging concept of fibrosis-aware precision medicine and propose a unifying framework to guide integrated therapeutic strategies. In the face of global climate change and rising environmental insults, a cross-organ perspective on fibrosis offers a timely and translationally relevant approach to addressing this growing burden on human health.

## 1. Introduction

Fibrotic remodeling has emerged as a key pathogenic process contributing to organ failure and increased patients’ mortality. It is now well established that 45% of deaths in developed countries can be attributed to mechanisms related to fibroproliferative disorders [1]. Current treatments only halt disease progression, thus leaving patients with major disability and impaired quality of life. Fibrosis refers to the aberrant accumulation of connective tissue components within an organ following an injury or persistent noxious stimuli. Regardless of the underlying cause, tissue damage triggers a cascade of cellular and molecular events that lead to the excessive deposition of extracellular matrix (ECM) components, resulting in tissue scarring, disruption of tissue architecture, and impairment of organ function [1,2]. Although fibrotic remodeling has traditionally been viewed through an organ-specific lens, it is now recognized that shared molecular mechanisms and cellular responses can affect virtually every organ system, including the skin and lung [3,4,5,6]. The concept of “cross-organ fibrosis” is, therefore, gaining attention, not only as a framework for understanding disease pathogenesis but also as a foundation for the development of broad-spectrum antifibrotic therapies [4]. Pulmonary fibrosis and cutaneous fibrotic conditions like systemic sclerosis and keloids serve as examples of chronic fibroproliferative disorders with growing prevalence and burden [4,5,7]. Fibrosis thus represents an urgent priority in current medical research and clinical practice [8,9].

Despite embryological differences in their epithelia, the lung and skin share mesoderm origins, functional parallels as barrier organs, and overlapping repair mechanisms mediated by conserved pathways, as TGF-β signaling [6,9]. Central to fibrosis is the persistent activation of fibroblasts into myofibroblasts, induced by mechanical stress, immune cues, and profibrotic cytokines [10,11].

Beyond genetic predisposition, environmental exposures—the exposome—play an equally crucial role in modulating disease trajectory [12,13,14]. As frontline barriers, the skin and lungs are particularly vulnerable to ultraviolet radiation, particulate matter, pathogens, and climate-related stressors such as pollution and thermal extremes, which can initiate or exacerbate fibrotic responses [15,16,17,18]. The skin responds to injury through a tightly regulated repair process, and impaired wound healing response leads to systemic sclerosis (SSc), hypertrophic scars, and keloids [19,20,21]. Similarly, the lungs are particularly susceptible to fibrotic remodeling due to recurrent exposure to airborne irritants, with idiopathic pulmonary fibrosis (IPF) representing a prototypical condition characterized by persistent alveolar epithelial injury and unchecked profibrotic signaling [5,22,23,24].

**Figure 1:**
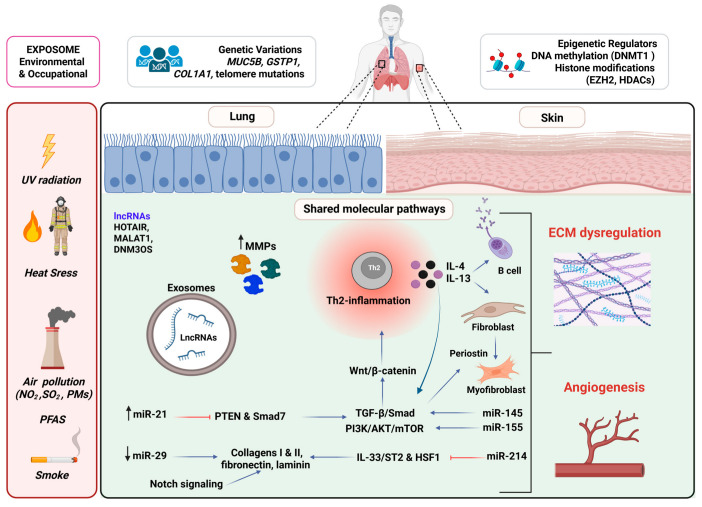
**Shared molecular pathways and environmental drivers of skin and lung fibrosis.** Environmental and genetic triggers (UV radiation, pollution, viral infection, telomere attrition, and risk alleles such as MUC5B) initiate epithelial or keratinocyte injury and immune activation. These upstream signals converge on profibrotic cytokines (IL-4/IL-13), autoimmune processes, and endothelial damage, which fuel fibroblast activation. Conserved signaling networks that drive chronic remodeling—including TGF-β/SMAD and non-SMAD cascades, Wnt/β-catenin stabilization, and Notch-driven epithelial–mesenchymal crosstalk—drive the transition of fibroblasts to myofibroblasts. Periostin further amplifies this profibrotic milieu by reinforcing ECM crosslinking and feedback to fibroblasts. Downstream, persistent ECM deposition (collagen or fibronectin) increases tissue stiffness and hypoxia, creating a vicious cycle of fibroblast reactivation. While organ-specific remodeling manifests as dermal thickening and immune infiltration in the skin, or alveolar injury and distal airway remodeling in the lung, both share a convergent fibrotic program. Epigenetic regulators—including dysregulated miRNAs (e.g., miR-21 upregulation or miR-29 loss), lncRNAs (HOTAIR, MALAT1, and DNM3OS), DNA methylation (DNMT1), and histone modifications (EZH2 and HDACs) fine-tune these responses. Synergistically, these pathways illustrate how environmental insults, genetic susceptibility, epigenetic regulation, and immune dysregulation coalesce into self-sustaining fibroinflammatory loops that underlie the common biology of skin and lung fibrosis. (**↑**: upregulation, **↓**: downregulation and ⟞: suppression) Created in BioRender. https://BioRender.com/83l0272 (accessed on 18 August 2025).

Recent advances have further highlighted the role of non-coding RNAs, particularly microRNAs (miRNAs), long non-coding RNAs (lncRNAs), and circular circRNAs, as pivotal regulators of fibrotic responses in both skin and lung tissues [25,26,27,28,29,30,31,32,33,34,35,36,37,38,39,40,41]. These RNA species orchestrate post-transcriptional fibrogenic networks involving TGF-β/Smad and inflammatory feedback loops. Notably, dysregulated miRNAs such as miR-21, miR-29, and miR-155 reveal a conserved epigenetic signature of fibrosis, offering potential for biomarker discovery and therapeutic intervention across organ systems [25,35,39] (Figure 1).

This review aims to examine the overlapping pathogenetic pathways that drive fibrosis in both the skin and the lung, particularly in the context of an increasingly complex exposome and a changing climate. By identifying convergent molecular circuits, regulatory RNAs, and environmental vulnerabilities, we aim to illuminate translational strategies that can inform cross-organ antifibrotic therapeutic approaches in an era increasingly defined by environmental instability and systemic health threats. 

## 2. Pathophysiology and Cellular Mechanisms

### 2.1. Shared Fibrotic Pathways in Skin and Lung Fibrosis

Fibrosis across tissues is driven by conserved molecular pathways that translate diverse insults—injury, infection, or environmental stress—into chronic remodeling. Central to these processes are TGF-β, Wnt/β-catenin signaling, ECM dysregulation, Interleukin (IL)-4/IL-13, and epithelial–mesenchymal interactions [42].

**TGF-β** is a master regulator of tissue repair and fibrogenesis, acting through multiple signaling cascades. TGF-β is secreted by various cell types—including platelets, macrophages, epithelial cells, and fibroblasts—and is sequestered in the ECM as part of a biologically inactive complex [43,44]. Once released from this latent form, active TGF-β binds to its type II receptor (TGF-βRII), which subsequently recruits and phosphorylates the type I receptor (TGF-βRI), triggering intracellular signal transduction. TGF-β receptor activation engages the SMAD-dependent (canonical) signaling network, which governs the transcriptional regulation of multiple genes, and the SMAD-independent (non-canonical) pathway, which influences key cellular processes such as polarity, cytoskeletal organization, and microRNA processing [43,44]. In both the skin and lung, aberrant TGF-β signaling sustains fibroblast activation and drives ECM accumulation (Figure 1).

The **canonical Wnt/β-catenin** signaling cascade also plays a critical role in fibrotic remodeling. Cytoplasmic β-catenin is targeted for degradation by the destruction complex that includes the tumor suppressors Axin and adenomatous polyposis coli (APC), the Ser/Thr kinases GSK-3 and CK1, protein phosphatase 2A (PP2A), and the E3-ubiquitin ligase β-TrCP [45,46]. Upon Wnt ligand (Wnt2, Wnt3a, or Wnt10b) binding to Frizzled (Fzd) receptors and LRP5/6 co-receptors, Disheveled (Dvl) recruits Axin to the membrane, disassembling the destruction complex [47,48]. This leads to β-catenin stabilization and nuclear translocation, where it binds T-cell factor TCF/ Lymphoid enhancer factor (LEF) transcription factors, displacing the repressive TLE/Groucho complex and recruiting transcriptional co-activators (CBP/p300, Pygo, BCL9, and BRG1) [46,49,50,51,52,53]. This initiates transcription of profibrotic genes, including collagens I/II, fibronectin, and α-SMA [51,54]. In cutaneous and pulmonary tissue fibroblasts, TGF-β can activate Wnt/β-catenin signaling through multiple mechanisms. Smad2/3–Smad4 complexes bind to promoters of Wnt ligand genes such as WNT2 and WNT10b, increasing their transcription [20,55]. Simultaneously, TGF-β represses transcription of Wnt antagonists, including DKK1 and sFRP1, thereby relieving inhibition of the Wnt/β-catenin signaling cascade [16,55]. The crosstalk between these pathways extends to non-Smad mechanisms like PI3K/Akt, which also contribute to signal integration. TGF-β activates PI3K/Akt signaling, which phosphorylates and inactivates GSK3β, indirectly stabilizing and translocating β-catenin in the nucleus [54]. Spatially, TGF-β and β-catenin signaling components frequently co-localize in fibrotic tissues. In idiopathic pulmonary fibrosis, nuclear β-catenin accumulation is observed in myofibroblasts that also express high levels of TGF-β1 [25,27]. Likewise, co-expression patterns have been documented in hypertrophic scar fibroblasts [20]. This coordinated activation accentuates a feed-forward loop in which TGF-β and Wnt signaling reinforce one another to drive persistent fibroblast activation and extracellular matrix deposition.

The **ECM** provides structural support and also regulates cellular phenotype and behavior through biochemical and mechanical signaling [56]. Dysregulation of the ECM is a hallmark and active contributor to fibrotic disease progression. Excessive ECM deposition or impaired degradation results in increased tissue stiffness, reduced organ compliance, and compromised function. These altered biomechanical properties further activate fibroblasts in a self-perpetuating cycle. Moreover, the ECM acts as a reservoir for latent profibrotic growth factors like TGF-β, which can be released and activated through mechanical stress or enzymatic remodeling, amplifying fibrogenic signaling [57,58]. Fibroblast activation is a highly conserved biological response to tissue damage, and once activated, they undergo phenotypic and functional changes. They acquire a contractile, secretory phenotype often characterized by the expression of alpha-smooth muscle actin (α-SMA), marking their transition into myofibroblasts. Notably, fibroblast activation protein (FAP) serves as a marker of activated stromal fibroblasts across various pathological conditions, while its expression is minimal or absent in quiescent fibroblasts under normal physiological conditions. As fibrosis advances, the accumulation of ECM increases tissue rigidity and induces hypoxia, both of which support ongoing fibrotic remodeling [9,59,60].

**IL-4 and IL-13**, principal mediators of type 2 immune responses, have recently garnered increasing attention for their roles in tissue regeneration and fibrosis; yet the molecular basis of these reparative pathways remains incompletely understood. While T-helper 2 (Th2) CD4^+^ T cells are the predominant source of IL-4 and IL-13, these cytokines are also produced by mast cells, eosinophils, and antigen-presenting cells [61,62]. IL-4 and IL-13 share a common receptor subunit, IL-4 receptor alpha (IL-4Rα), and signal through the Janus kinase (JAK)/signal transducer and activator of transcription 6 (STAT6) pathway to orchestrate transcriptional programs that stimulate fibroblast migration and enhance the synthesis of collagen and other ECM components. Furthermore, IL-4/IL-13 signaling upregulates expression of profibrotic genes, including type I collagen and TGF-β itself, creating a feedback loop that amplifies fibrosis pathogenesis [63,64].

**Notch signaling** is a key profibrotic pathway active in both skin and lung fibrosis. In cutaneous fibrosis (e.g., keloids, hypertrophic scars, and scleroderma), Notch1, Jagged1, and NICD are upregulated in keratinocytes and fibroblasts, contributing to fibroblast activation and ECM deposition [21,65,66]. Notch also regulates epidermal homeostasis and keratinocyte–fibroblast crosstalk, while ECM stiffness sustains fibroblast activation [20,21,67,68,69]. In pulmonary fibrosis, Notch signaling is similarly elevated in myofibroblasts and airway epithelium, driving tissue remodeling and alveolar distortion [65,70]. A shared mechanism involves Notch–TGF-β crosstalk, which promotes Smad3 phosphorylation, and EMT [71,72]. Inhibition of Notch (e.g., γ-secretase inhibitors or Notch1 deletion) reverses fibrosis in both organs [73,74]. Notch also cooperates with Wnt and Hedgehog pathways, and combined inhibition offers synergistic antifibrotic effects with improved tolerability [9].

**Periostin**, a matricellular protein induced by IL-4 and IL-13, is a potent modulator of fibroblast activity and ECM crosslinking [75,76]. It is highly expressed in fibrotic skin and lung tissues, including scleroderma plaques, IPF lesions, and HS tracts [77,78]. In the lung, periostin enhances fibroblast recruitment and collagen production, while its serum levels correlate with IPF progression and therapeutic response to nintedanib. In the skin, elevated periostin levels have been linked to dermal stiffness and fibrosis progression in SSc and keloids [79,80,81]. This supports periostin’s emerging role as both a biomarker and therapeutic target across fibrotic diseases [82,83,84].

### 2.2. Fibroinflammatory Remodeling in Lung and Skin Fibrosis

Fibroinflammatory remodeling represents a central pathological process in chronic lung and skin fibrotic diseases, where the interplay of sustained inflammation and progressive fibrosis leads to irreversible tissue architecture disruption. A shared hallmark characteristic of both skin and lung fibrosis is the persistent activation of fibroblasts into contractile, ECM-secreting myofibroblasts. This phenotypic switch is maintained through synergistic signaling by TGF-β/SMADs, IL-4/IL-13 via JAK/STAT6, and Wnt/β-catenin pathways [16,25,67]. Additionally, ECM stiffness acts as both a consequence and amplifier of fibrosis through mechanotransduction pathways, reinforcing the myofibroblast phenotype and sustaining fibrotic remodeling [27,69].

Environmental exposures contribute significantly to fibroinflammatory responses. In the skin, chronic UV radiation and other barrier-disruptive insults trigger oxidative stress, keratinocyte damage, and immune activation. In the lung, PM, smoke, and other airborne pollutants initiate alveolar epithelial injury and inflammation [15,17,18,28]. These exposomal factors lead to alveolar epithelial cell apoptosis in genetically predisposed individuals (short telomeres or MUC5B mutations); disruption of the alveolar epithelial cell membrane; and crosstalk between structural cells, including apoptotic alveolar epithelial cells and fibroblasts. This interaction involves secretion of numerous profibrotic signaling molecules, including TGF-β. TGF-β promotes fibroblast recruitment, proliferation, and survival; epithelial-to-mesenchymal transition; fibroblast-to-myofibroblast conversion; and secretion of other profibrotic signals, leading to excess collagen production and deposition [19]. It is important to underline that within the remodeled fibrotic lung, distal (smaller) airways begin to resemble proximal (larger) airways in terms of their cellular composition and structure. This process, called proximalization of distal airways, is characterized by the loss of normal distal airway characteristics and the adoption of features more typical of proximal airways, including loss of Terminal Airway-Enriched Secretory Cells (TASCs) and enrichment with aberrant basaloid cells [85,86]. These cells surrounding fibroblastic foci represent a recently identified epithelial cell population that exhibits a mix of epithelial and mesenchymal cell characteristics and are thought to play a major role in lung fibrosis development and progression [87].

Importantly, chronic inflammation involving cytokines like IL-6 and TNF-α further sustains the fibroblast activation loop in both organs [16,25,67]. These cytokines not only sustain immune cell infiltration but also prime fibroblasts toward a pro-inflammatory and fibrogenic status. Urbanization-related exposures and pollution have been shown to exacerbate type 2 inflammation via epigenetic alterations [88,89,90]. In the lung, repeated alveolar epithelial injury caused by environmental insults, autoimmunity, or idiopathic mechanisms (as in IPF) leads to persistent immune activation and an inflammatory milieu rich in TGF-β, IL-6, and IL-13. These cytokines drive fibroblast-to-myofibroblast differentiation, ECM accumulation, and the progressive distortion of alveolar architecture, ultimately impairing compliance and gas exchange [16,17,91]. In the skin, similar fibrotic cascades occur, particularly in SSc and localized scleroderma, where keratinocyte injury, autoantibodies, and mechanical stress lead to dermal immune infiltration, endothelial damage, and fibroblast activation. These fibroblasts increasingly adopt profibrotic and pro-inflammatory phenotypes, contributing to dermal thickening, loss of elasticity, stiffness, and permanent scarring [92,93,94].

Emerging evidence suggests that fibroinflammatory remodeling is perpetuated by a self-sustaining loop involving persistent low-grade inflammation (inflammaging) in both aged skin and repeatedly injured tissue [16,92,95]. Fibroblast heterogeneity includes subsets with distinct inflammatory, ECM-producing, or senescent phenotypes, including CTHRC1 high pathogenic fibroblasts [96,97]. Furthermore, cellular senescence and accumulation of senescence-associated secretory phenotype (SASP) factors amplify inflammation and matrix deposition [98]. Additionally, loss of epithelial/epidermal integrity reduces antifibrotic signaling and enables persistent immune-fibroblast crosstalk and tissue remodeling [17,93,99]. Moreover, aberrant repair mechanisms fail to resolve inflammation and dysregulated tissue regeneration [100]. Emerging data also support a role for epigenetic regulators, particularly non-coding RNAs (miRNAs and lncRNAs), which modulate fibrotic gene expression post-transcriptionally and constitute a shared regulatory layer across fibrotic tissues (Figure 1).

Understanding fibroinflammatory remodeling as a shared process opens opportunities for cross-organ antifibrotic strategies targeting core pathways such as TGF-β, JAK/STAT, and IL-13/IL-4 signaling.

### 2.3. Clinical Characteristics of Skin and Lung Fibrosis: Focus on Systemic Sclerosis

Skin and lung fibrosis frequently co-occur in systemic autoimmune diseases, where immune dysregulation drives fibrotic remodeling across organs. Clinically, this manifests as cutaneous thickening, sclerotic skin changes, and interstitial lung involvement—features that significantly contribute to patient morbidity.

**Figure 2:**
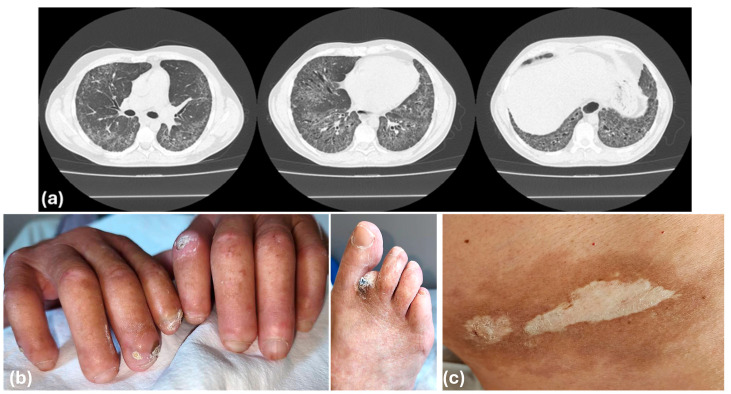
(**a**) High-resolution computed tomography (HRCT) scan of the chest in a patient with systemic sclerosis-associated interstitial lung disease (SSc-ILD). The scan demonstrates imaging features consistent with a fibrotic non-specific interstitial pneumonia (NSIP) pattern, including bilateral, basal-predominant ground-glass opacities with relative subpleural sparing, reticulation, and traction bronchiectasis. Additionally, a dilated esophagus is visible, a common extrathoracic manifestation of systemic sclerosis. (**b**) Scleroderma in the fingers, toes, and (**c**) in the abdominal area with characteristic central fibrosis. (Courtesy of the archive of Prof. Argyris Tzouvelekis and Dr. Katerina Grafanaki).

Dual-organ fibrosis is observed in systemic sclerosis (SSc), Systemic Lupus Erythematosus, Mixed Connective Tissue Disease, Antisynthetase Syndrome, Sarcoidosis, Rheumatoid Arthritis (typically in later disease stages, RA-ILD), and in fibrosis induced by certain medications (e.g., bleomycin) or infections like *Mycobacterium abscessus* [101,102,103]. Common pathways involve TGF-β signaling, fibroblast activation, and chronic inflammation. While environmental and mechanical insults may initiate fibrogenesis, persistent autoimmunity—independent of overt trauma—can likewise serve as a primary initiator and driver of fibrosis, where endothelial injury, autoantibodies, and chronic cytokine signaling sustain fibroblast activation. However, this review will narrow its scope to shared fibrotic mechanisms between skin and lung tissues in SSc, deliberately excluding a broader discussion on systemic autoimmunity (Figure 2).

SSc is a chronic autoimmune disease characterized by fibrosis, vasculopathy, and immune dysregulation [104]. Whole-genome expression profiling has identified four intrinsic molecular subsets: inflammatory, fibroproliferative, normal-like, and limited [105,106,107]. These subsets differ in immune, fibrotic, and cell-cycle activity and help explain variability in treatment responses [108]. Early disease is associated with IFNα signaling, showing strong expression, while TGFβ signaling dominates the fibroproliferative and inflammatory subsets. Furthermore, the fibroproliferative subset was most strongly associated with PDGF signaling, whereas the inflammatory subset was associated with activation of innate immune pathways such as TLR signaling upstream of NF-κB. In contrast, the limited and normal-like subsets showed no associations with fibrotic and inflammatory mediators such as TGFβ and TNFα [109]. Clinical trials that overlook this heterogeneity may underestimate drug efficacy, whereas retrospective analyses reveal differential treatment responses across subsets [110].

SSc-ILD is characterized by distinct radiologic patterns—most notably non-specific interstitial pneumonia (NSIP) and less commonly usual interstitial pneumonia (UIP)—within the clinical framework of systemic sclerosis. Prevalence estimates suggest that ILD occurs in 30–50% of SSc patients, though autopsy series indicate subclinical fibrosis may be present in over 75% of cases [101,102,103]. Importantly, pulmonary fibrosis is the leading cause of mortality in SSc, accounting for approximately 20–40% of deaths [111]. ILD may develop at any stage of the disease, irrespective of duration, emphasizing the need for early baseline and longitudinal monitoring using high-resolution computed tomography (HRCT), pulmonary function tests (PFTs), and symptom-based assessments such as lung auscultation for the pathognomic end-inspiratory velcro-type crackles [112,113]. Risk factors for progression include diffuse cutaneous SSc, anti-topoisomerase I antibodies, male sex, African ancestry, and elevated inflammatory markers such as CRP and ESR [114,115].

The pathogenesis of fibrosis in both skin and lung in SSc shares core mechanisms centered on fibroblast dysregulation, aberrant tissue repair, and chronic inflammation. In the skin, fibrosis often stems from abnormal wound healing and autoimmunity, while in the lung, it parallels ILD development.

SSc demonstrates the complexity of skin fibrosis, which involves vasculopathy, immune dysregulation, and fibrotic remodeling in a multi-organ context. Notably, the fibrotic skin changes in SSc serve as both a clinical hallmark and a model for studying fibroblast heterogeneity and autoimmunity-driven fibrosis [15]. Autoreactive B cells contribute to SSc via autoantibody production, pro-inflammatory cytokines (IL-6 and TGF-β), and fibroblast activation [110]. Autoantibodies such as anti-topoisomerase I and anti-centromere directly contribute to pathogenesis by targeting endothelial and stromal cells, initiating an inflammatory cascade that ultimately leads to fibrotic remodeling [116,117,118,119]. Early microvascular injury initiates a sequence involving hypoxia, recruitment of inflammatory cells, and sustained TGF-β–mediated fibroblast activation [120,121]. Cytokines like IL-6 and IL-13 play central roles at this immune–fibrotic interface, reinforcing a cycle of chronic fibrosis [15,20,67,122].

## 3. Epigenetic Regulation in Skin and Lung Fibrosis

Fibrotic diseases are shaped by both genetic and environmental factors, but epigenetic modifications such as DNA methylation, histone modifications, and non-coding RNA circuitry play critical roles in profibrotic cytokine signaling [123]. In both organs, lncRNAs modulate fibrogenesis and influence cellular behavior via chromatin remodeling, ceRNA networks, and transcriptional regulation (Figure 1).

### 3.1. Epigenetics of Skin Fibrosis

In SSc, aberrant DNA methylation patterns have been observed in dermal fibroblasts, leading to dysregulation of key fibrogenic genes such as *COL1A1*, *TGFB1*, and genes involved in the Wnt and TGF-β signaling pathways [124,125,126]. Notably, genome-wide methylation studies have revealed both shared and subset-specific methylation changes in diffuse vs. limited SSc, suggesting distinct epigenomic trajectories within the disease spectrum [126]. African American patients with SSc display a differential methylation landscape in skin fibroblasts, underscoring the role of ancestry-specific epigenetic regulation in fibrotic susceptibility [127]. Moreover, environmental insults such as ionizing radiation can induce fibrosis through epigenetic silencing of regulatory genes. For instance, methylation-dependent repression of SLC39A9 (ZIP9) enhances TGF-β signaling and fibroblast activation, providing a direct mechanistic link between environmental triggers and epigenetic fibrotic reprogramming [128]. Histone modifications also play a central role in fibrotic gene expression. In bleomycin-induced skin fibrosis mice, intraperitoneal administration of the histone deacetylase (HDAC) inhibitor Trichostatin A (TSA) at 0.5 μg/g/day significantly attenuated dermal ECM accumulation with no obvious toxic effects. After 4 weeks, histological analysis of skin in TSA-treated mice (n = 10) exhibited a 16% (*p* < 0.05) reduction in dermal thickness compared with controls (n = 14). These results underscore the therapeutic potential of HDAC inhibition as an epigenetic strategy to reverse ECM deposition in fibrosis in vivo [129].

Long non-coding RNAs (lncRNAs), typically >200 nucleotides in length, have emerged as dynamic regulators of fibrosis, functioning through diverse mechanisms including chromatin remodeling, miRNA sponging, and transcriptional regulation [130,131]. In skin fibrosis, several lncRNAs have been identified as critical modulators of fibroblast proliferation, ECM deposition, and migration. SSc myofibroblasts and skin biopsies show elevated HOTAIR, an lncRNA that recruits EZH2 to induce H3K27me3, suppress miR-34a, activate NOTCH, and upregulate GLI2, which drives profibrotic marker expression [132]. EZH2 inhibition reverses these effects [133]. Other profibrotic lncRNAs include *LINC00525*, *LINC01711*, and *uc003jox.1*, which regulate TGF-β or PI3K/AKT signaling pathways *via* competing endogenous RNA (ceRNA) mechanisms [134,135,136,137]. For example, the SNHG1/miR-320b/CTNNB1 axis modulates fibroblast migration during keloid formation, while *GNAS-AS1* knockdown reduces keloid growth *via* the miR-188-5p/RUNX2 pathway [138,139]. Additionally, lncRNAs participate in organ-wide fibrosis networks, are conserved, and potentially relevant to skin fibrosis as well [140]. Age-related epigenetic drift and lncRNA modulation also link aging to fibrogenesis [141]. Their disease and tissue specificity make lncRNAs promising biomarkers and therapeutic targets [130].

### 3.2. Epigenetics of Lung Fibrosis

IPF and other interstitial lung diseases (ILDs) and environmental insults (e.g., smoking or pollution) initiate disease, while epigenetic dysregulation contributes to progression and therapeutic resistance [2]. Studies have revealed widespread DNA methylation abnormalities in IPF lungs, with hypermethylation of antifibrotic genes including *PTEN* and *FOXO3*, and hypomethylation of profibrotic mediators such as TGF-β pathway components [142,143,144]. Single-cell and bulk epigenomic studies confirm that DNA methylation is not only globally dysregulated in IPF but is also cell-type specific, particularly affecting epithelial cells and myofibroblasts [143,145].

Importantly, loss of function in epigenetic regulators exacerbates fibrosis. Deficiency of DNA methyltransferase 3B (DNMT3B) in myeloid cells enhances macrophage-driven fibrogenesis, which suggests that epigenetic enzymes tightly regulate the immune-fibrotic crosstalk [146]. Moreover, histone methyltransferases such as EZH2 also contribute to fibrotic gene silencing or activation, providing mechanistic links between chromatin remodeling and fibroblast persistence [145,147].

In IPF, lncRNAs such as MALAT1, DNM3OS, and TP53TG1 are differentially expressed in IPF lung tissues, are associated with disease severity, and correlate with fibrotic gene expression of *PTEN* and *FOXO3* [144,148]. Others like ABCE1-5 and MIR205HG regulate fibroblast activation and ECM production by interacting with epithelial markers like KRT14, or by modulating immune mediators like IL-33 via Alu elements, respectively [149,150]. LncRNAs also regulate fibrogenic pathways by modulating epigenetic regulators. FEZF1-AS1 upregulates EZH2 and promotes EMT and ECM gene expression via the miR-200c-3p/ZEB1 axis [147]. Similarly, ANRIL contributes to fibroblast activation by sponging let-7d-5p, upregulating *TGFBR1* levels in TGF-β1-stimulated lung fibroblasts. This depicts the lncRNA–miRNA–mRNA competitive endogenous RNA (ceRNA) axis in fibrosis regulation [151]. LncRNA CBR3-AS1 functions as a central node in the CBR3-AS1/miR-29/FIZZ1 axis, which integrates miRNA and cytokine signaling. By sequestering miR-29, it regulates FIZZ1 (RELMα, Resistin-like molecule α1), a profibrotic cytokine, to modulate ECM remodeling, highlighting its potential as a therapeutic target [152].

LncRNA-mRNA co-expression and regulatory analyses confirm that lncRNAs operate as upstream epigenetic regulators of fibroblast phenotype and behavior at multiple levels [153,154]. Furthermore, lncRNAs are actively secreted in extracellular vesicles (exosomes), suggesting their role as systemic mediators and potential circulating biomarkers of disease progression and treatment response [155].

## 4. MicroRNAs in Skin and Lung Fibrosis: Shared Mechanisms and Molecular Pathways

In both skin and lung, microRNAs (miRNAs) play key regulatory roles in the transcriptional and post-transcriptional control of profibrotic and antifibrotic pathways. A growing body of evidence from transcriptomic, mechanistic, and translational studies suggests that certain miRNAs serve as shared epigenetic regulators in fibrosis across different tissues, particularly miR-21, miR-29, miR-155, miR-145, and miR-214 (Figure 1).

A profibrotic driver in skin and lung is mir-21, one of the most consistently upregulated miRNAs in fibrotic diseases. In hypertrophic scar and keloid fibroblasts, miR-21 expression is elevated ~2.5–4.2-fold compared with normal skin (*p* < 0.01), driving TGF-β/Smad and PI3K/Akt activation by suppressing Smad7 and PTEN [37,38,39,40,41], while in plasma from 88 IPF patients, miR-21 levels were elevated approximately 2-fold compared to healthy controls (*p* < 0.001) [156]. A recent study revealed that METTL3-mediated m6A RNA methylation was upregulated in fibrotic mouse lungs, promoting aberrant differentiation of lung-resident mesenchymal stem cells into myofibroblasts via the miR-21/PTEN pathway. Inhibition of METTL3 or miR-21, or overexpression of PTEN, reversed this effect, uncovering a novel mechanism in pulmonary fibrosis and potential therapeutic targets [157]. Furthermore, in bleomycin-induced mouse models (1.5 U/kg; 10 mice per group), treatment with anti-miR-21 oligonucleotides (2 mg/kg) reduced collagen and fibronectin mRNA and protein levels by ~30–70% (*p* < 0.001) and decreased the Ashcroft score by ~4 units (*p* < 0.05), indicating a significant reduction in pulmonary fibrosis severity [158]. Thus, targeting miR-21 via anti-miR strategies has been shown to alleviate fibrosis in both contexts.

On the other hand, the miR-29 family (miR-29a, miR-29 b, and miR-29c) is markedly downregulated in skin and lung fibrosis, functioning as a master suppressor of ECM genes, including collagens I and III and hydroxyproline. In fresh skin biopsies from SSc patients versus healthy controls (n = 5 of each group), miR-29 levels were reduced by 40–55% (*p* < 0.05). In the same study, overexpression of miR-29a in SSc fibroblasts decreased type I and III collagen mRNA by about 65% (*p* < 0.0003) and 35–45% (*p* < 0.0006) in the protein level, whereas knockdown in normal fibroblasts increased them at the same level, indicating direct regulation of collagens. Similarly, in the bleomycin-induced skin fibrosis mouse model (n = 10 mice per group), the miR-29 family was also downregulated by about 30–70% (*p* < 0.02), and inhibition of PDGF-B and TGFβ pathways with imatinib (150 mg/kg/day for 3 weeks and n = 8 mice per group) restored its expression in vivo [159]. Regarding IPF, miR-29 family expression was suppressed by ~2-fold (*p* < 0.01) in bleomycin-induced lung fibrosis mice (n = 5/group; 1.5 U/kg). Sleeping Beauty–mediated miR-29 delivery reversed fibrosis and inflammation, demonstrated by a ~2-fold increase (*p* < 0.01) in hydroxyproline and collagens I and III [160]. A novel study has also showed that in a bleomycin-induced pulmonary fibrosis mouse model (0.01 U/kg for 3 days), intranasal delivery of 60 μmol of a single-stranded miR-29b mimic, Psh-match, led to a significant improvement in fibrosis, reducing the Ashcroft score to ~2 units (*p* < 0.05), hydroxyproline to 10 units, and collagen I to 0.6-fold (*p* < 0.05). Notably, miR-29b Psh-match did not activate Toll-like receptor signaling pathways, suggesting a safer profile for clinical applications [161]. Regarding skin fibrosis, in a phase 1 clinical trial with 47 subjects, intradermal administration of the miR-29 mimic Remlarsen into intact or incisional skin (6 doses over 2 weeks) significantly reduced collagen and metalloproteinase mRNA expression by 2–6-fold (*p* < 0.05), and histological analysis of biopsies showed approximately a 50% reduction in fibroplasia in depth and area at wound sites, demonstrating an in vivo antifibrotic effect [162]. Synthetic miR-29 mimics show robust antifibrotic activity in preclinical skin and lung models, supporting their promise as therapeutic agents.

A modulator of the inflammatory microenvironment that fuels fibrosis is miR-155. In skin, it enhances fibroblast proliferation and ECM production by targeting HIF-1α and modulating PI3K/AKT signaling [18,163,164,165,166]. In lung fibrosis, particularly in systemic sclerosis-associated interstitial lung disease, miR-155 is upregulated and promotes Th17 responses and macrophage activation, exacerbating fibrotic remodeling [167]. Myofibroblast differentiation across tissues is promoted by mir-145 through targeting transcriptional repressors, like KLF4 and ZEB1. In recessive dystrophic epidermolysis bullosa (RDEB) skin fibroblasts, miR-145-5p enhances fibrotic features [168]. In lung fibroblasts, miR-145 facilitates TGF-β-driven differentiation to myofibroblasts and ECM protein production [169,170,171]. Interestingly, miR-214 appears to play a dual role in ECM regulation, where it targets the IL-33/ST2 axis in the skin and the HSF1 pathway in the lung, contributing to ECM turnover and fibroblast activation [23,172].

## 5. The Exposome Driving Skin and Lung Fibrosis

The exposome refers to the totality of environmental exposures, including chemical, physical, biological, and psychosocial stressors, that individuals encounter across the lifespan, beginning in utero, and how these exposures influence health outcomes [173,174]. This concept offers a framework to understand the multifactorial origins of complex diseases such as skin and lung fibrosis, which, despite differing anatomically, share common exposomal drivers, molecular mechanisms, and clinical trajectories. Due to their barrier functions and direct environmental interface, both organs are vulnerable to external insults. Air pollution, ultraviolet (UV) radiation, and occupational hazards are well-established drivers of oxidative stress, epithelial injury, chronic inflammation, and premature aging, all of which are key mechanisms underlying fibrogenesis [175,176,177]. Herein, we outline the key exposomal factors to fibrosis, underlying mechanisms, and high-risk populations.

### 5.1. Climate Change and Pollution

Climate change variables, including increased UV radiation, rising temperatures, and humidity shifts, have been linked to barrier dysfunction, inflammaging, and fibrosis via pathways involving TGF-β signaling, cellular senescence, microbiome dysbiosis, and immune dysregulation [178,179,180]. Rising global temperatures are often coupled with air pollution, acting as potent stressors that impair epithelial integrity, trigger chronic inflammation, and promote persistent tissue remodeling (Figure 1).

In the skin, chronic heat exposure compromises epidermal integrity, disrupts hydration, and alters ECM protein conformation. These effects promote cellular senescence, chronic low-grade inflammation and ultimately dermal fibrosis, particularly in aged or exposed skin [179,180,181]. Increased mechanical stiffness of the skin, due to photoaging or scarring, activates the Piezo1-Wnt2/Wnt11-CCL24 mechanoresponsive pathway, amplifying fibroinflammation and collagen deposition [182]. Additionally, thermal stress impairs skin hydration and barrier function, predisposing residents to dermatitis, eczema, and potentially dermal fibrosis, especially in vulnerable populations such as the elderly and outdoor workers, to conditions like eczema and dermal fibrosis [183,184].

In the lungs, heat enhances the reactivity and penetration of inhaled pollutants, including ozone, volatile organic compounds (VOCs), and PMs [185,186]. These interactions damage alveolar epithelial cells and amplify profibrotic signaling pathways [13,187,188]. Heatwaves have been associated with exacerbations of asthma, COPD, and ILDs, especially in older adults and those with preexisting respiratory conditions [189]. Moreover, heat shock proteins (HSPs) are upregulated in response to thermal and oxidative stress. Although initially protective, sustained HSP expression—especially HSP70 and HSP90—enhances TGF-β signaling, stabilizes the myofibroblast phenotype, and drives both cutaneous and pulmonary fibrogenesis [190,191,192].

Per- and polyfluoroalkyl substances (PFAS), including perfluorooctanoic acid (PFOA), are highly persistent environmental toxicants increasingly linked to fibrotic alterations in both lung and skin. These “forever chemicals” accumulate in biological tissues, where they disrupt epithelial homeostasis, immune balance, and ECM remodeling. In pulmonary systems, PFAS impair alveolar integrity and elicit pro-inflammatory responses that precede fibrogenesis. Experimental data confirm PFAS-induced oxidative stress, inflammasome activation, and cytokine dysregulation in lung epithelial and immune cells [193,194]. Additionally, PFAS-bound PM can exacerbate pulmonary toxicity, amplifying epithelial damage and fibrotic signaling [195]. Epidemiological findings from the ESPINA study correlate elevated serum PFAS with reduced lung function extending from adolescence into adulthood [196].

In the skin, although direct human data on PFAS-driven fibrosis are limited, toxicokinetic modeling supports dermal absorption and systemic distribution, particularly of PFOA [197,198,199]. PFAS are known to impair barrier function, keratinocyte differentiation, and immune signaling—all pathways implicated in fibrotic skin remodeling [174,200,201]. Chronic or occupational exposure may promote fibroblast activation and ECM deposition, resembling pulmonary mechanisms. Their bioaccumulation in cutaneous tissues disrupts keratinocyte–fibroblast crosstalk and perpetuates oxidative and inflammatory signaling, suggesting a shared fibrotic trajectory across organs.

Furthermore, airborne pollutants—including PM, polycyclic aromatic hydrocarbons (PAHs), nitrogen dioxide (NO_2_), ozone, and sulfur dioxide—promote oxidative stress, mitochondrial damage, and accelerated skin aging, contributing to dermal fibrosis [202,203,204]. PM_2.5_ in particular, with their small size, can penetrate the epidermis or act systemically, activating fibroblasts and worsening morphea, post-burn scarring, and atopic dermatitis [181,205,206,207,208]. PFAS compounds bioaccumulate in skin, disrupting immune and fibroblast-keratinocyte signaling and promoting fibrotic remodeling [197,209].

In the lungs, air pollution causes alveolar epithelial injury, oxidative stress, and dysregulated repair. Exposure to PM_2.5_, PM_10_, NO_2_, and ozone is linked to increased mortality, radiographic progression, and accelerated decline in patients with fibrotic ILDs, including IPF [210,211,212,213,214,215,216]. Notably, prolonged exposure to higher concentrations of atmospheric pollutants and tobacco smoke appears to independently contribute to acute exacerbations of idiopathic pulmonary fibrosis (IPF) and idiopathic interstitial pneumonias [184,214,217].

Beyond direct injury, pollution drives epigenetic modifications that reshape transcriptional programs linked to immune dysregulation and fibrosis [218,219,220,221,222]. For instance, PM exposure upregulates DNMT1 and induces aberrant DNA hypermethylation at fibrogenic loci such as the COL1A1 promoter, enhancing excessive collagen deposition [223,224]. In IPF patients, PM_2.5_ exposure correlates with altered global DNA methylation patterns [225]. Additionally, silica and dust exposures act via HDAC4/Smad2/3 signaling, reinforcing the fibrotic epigenome [226]. Importantly, genetic polymorphisms such as GSTP1 variants exacerbate pollutant-induced oxidative stress, intensifying fibrosis progression [227]. Notably, although both UV radiation and air pollution are independently recognized as risk factors in fibrotic lung disease, their potential synergistic effects with genetic predispositions such as telomerase mutations in IPF remain insufficiently characterized [228]. Overall, these insights point toward a gene–environment interaction paradigm, where inherited vulnerabilities amplify the impact of pollutant-induced epigenetic signatures, culminating in persistent fibrotic remodeling [220].

### 5.2. UV Radiation, Wildfires, and Burns

#### 5.2.1. UV Radiation-Induced Fibrosis

Chronic UV exposure accelerates skin aging and fibrosis through both direct and indirect mechanisms. Ultraviolet A (UVA) and UVB rays induce reactive oxygen species (ROS), DNA damage, and mitochondrial dysfunction, triggering matrix metalloproteinase (MMP) activation, leading to degradation of structural collagens (types I and III), ECM dysregulation, remodeling, and dermal thickening [30,31,32,229]. Epidermal stem cells experience cumulative DNA damage under chronic UV exposure, leading to senescence and impaired regenerative capacity [31]. Furthermore, UV-induced skin injury leads to selective loss of papillary fibroblasts and expansion of profibrotic reticular lineages, alongside recruitment of immune cells that perpetuate the fibrotic niche [34].

Radiation-induced pulmonary and cutaneous fibrosis is a delayed complication of radiotherapy, primarily mediated by microvascular damage, persistent myofibroblast activation, and cell death pathways such as apoptosis and ferroptosis [176,230,231]. The incidence of radiation-induced lung fibrosis ranges from 16% to 28%, while up to one-third of patients undergoing radiotherapy for breast cancer or chest wall tumors may develop dermal fibrosis. These complications represent distinct clinical syndromes, characterized by a spectrum of symptoms that can significantly impair quality of life and long-term outcomes. Pulmonary manifestations include chronic cough, pleuritic chest pain, dyspnea, pulmonary hypertension, and reduced respiratory capacity, whereas cutaneous involvement may present with alopecia, skin induration, and ulceration [231,232,233,234,235]. Protective strategies targeting oxidative stress, such as *Botryocladia leptopoda* extracts, may reduce UV-induced scarring and enhance collagen synthesis [32].

#### 5.2.2. Wildfires and Thermal Injury

With extreme temperatures come increasingly destructive wildfires, which represent a dual environmental threat, combining heat and inhalational exposures. These events release ROS, hydrocarbons, and PMs that damage the epithelium, promote inflammation, and initiate fibrotic cascades in the lung [13,187,188].

Thermal injuries, such as burns, represent another major fibrotic driver. Cutaneous burn injury elicits a profound inflammatory response, marked by acute tissue necrosis, release of cytokines like IL-6 and TGF-β, and fibroblast-to-myofibroblast transition [236,237]. This leads to excessive ECM accumulation, angiogenesis, and often hypertrophic scarring (HSCs) and keloid formation. These fibrotic responses severely affect quality of life and remain a major clinical challenge. HSCs and keloids, though mechanistically overlapping, exhibit distinct histologic features. [36]. The HSR, notably HSF1, is overexpressed in keloid fibroblasts, driving collagen I/III and α-SMA expression. Inhibition of HSF1 has been shown to reverse the fibrotic phenotype, suggesting that the HSR–TGF β axis is a critical driver of thermally induced fibrosis [35,237].

Additionally, radiation-induced skin fibrosis (RISF) shares mechanistic similarities with heat injury. Recent studies implicate angiotensin II signaling in RISF pathogenesis, suggesting that renin–angiotensin system blockade may offer antifibrotic therapeutic potential [33].

Burn injuries frequently co-occur with inhalation damage, which significantly contributes to morbidity and worsens clinical outcomes. Smoke inhalation during fire-related events increases alveolar–capillary permeability and induces oxidative stress, initiating cytokine storms rich in IL-1β, IL-6, and TGF-β, which collectively drive fibrotic lung remodeling. Thermal damage to the airway epithelium disrupts surfactant homeostasis and initiates alveolar inflammation, often progressing to acute lung injury (ALI) or acute respiratory distress syndrome (ARDS) [238,239]. In genetically or immunologically predisposed individuals, these acute injuries may transition into chronic pulmonary fibrosis [13,187,188]. Repeated or high-intensity exposures, such as those experienced during wildfires, are associated with increased rates of pulmonary fibrosis, ILD exacerbations, and airway remodeling [240,241].

Collectively, the overexpression of HSPs in both skin and lung tissues under thermal stress directs towards a shared fibrogenic pathway. These findings support the concept of a unified thermal exposome as a model in which heat-related insults across organs activate overlapping molecular pathways that drive multi-organ fibrotic remodeling.

### 5.3. Occupational Exposome

Climate change and urbanization have increased global exposure to environmental and occupational exposure to heat stressors, air pollutants, and industrial chemicals. Occupational ILDs are now recognized as a distinct subgroup of pulmonary fibrosis notable for their relatively high prevalence, preventable etiology, and the need for specific management strategies. Timely diagnosis hinges on a detailed occupational exposure history, geospatial exposure assessment, and prompt removal from a hazardous environment to prevent disease progression. Classic occupational hazards such as silica, asbestos, and organic solvents are strongly associated with SSc-associated ILD and occupational pulmonary fibrosis [242,243,244]. SSC with marked fibrosis of both skin and lung shows strong geographic clustering in polluted areas and populations exposed to heavy metals and silica [245,246,247,248].

Among the best-characterized work-related ILDs are silicosis, coal workers’ pneumoconiosis, asbestosis, chronic beryllium disease, and certain forms of hypersensitivity pneumonitis, all of which arise from repeated or prolonged inhalation of specific occupational antigens [249,250]. Large-scale epidemiological studies from Europe and the U.S. report increased IPF risk linked to cumulative exposure to dust and industrial chemicals, independent of smoking status [244,251,252]. A recent meta-analysis confirmed elevated IPF risk with exposure to vapors, gases, dusts, and fumes (VGDF), highlighting occupational exposure as a major, yet frequently underrecognized, etiological factor [252].

Firefighters represent a high-risk group due to their repeated exposure to heat, smoke, toxic gases, and combustion-derived particulates. These exposures place them at the epicenter of thermal and chemical injury. Chronic inhalation of airborne irritants, including particulate matter, volatile organic compounds, and carbonaceous aerosols, has been linked to airway remodeling, COPD, and ILD [241,253]. Data from the World Trade Center Health Registry confirmed an increased incidence of pulmonary fibrosis among responders, suggesting that acute high-intensity exposures may initiate persistent fibrotic remodeling [240]. Furthermore, IPF has been documented in this population, often following a latent period of subclinical inflammation [254,255].

In addition to inhalational risks, firefighters also face significant cutaneous exposure. Simultaneously, prolonged use of heat-retaining protective gear can impair thermoregulation and skin ventilation, exacerbate barrier dysfunction, and contribute to eczema, contact dermatitis, thermal burns, and delay wound healing, all of which can result in fibrotic scarring [256]. These dermal insults, compounded by chronic exposure to chemical irritants, contribute to skin inflammation and may drive systemic fibrotic responses in predisposed individuals [237,257]. In diseases like SSc, chronic inflammation and fibrotic remodeling concurrently affect both skin and lung [258,259]. The cumulative dermal and respiratory burden in firefighting is now recognized as a distinct occupational exposome, demanding tailored preventive and clinical strategies [240,241].

Other occupational groups at risk include workers in mining, shipbuilding, construction, agriculture, military service, and industrial manufacturing. These individuals face long-term exposure to silica dust, metal particles, organic solvents, smoke, and repeated heat stress [260,261,262]. Meta-analyses confirm increased IPF incidence in those employed in farming, construction, and manufacturing, with VGDF exposure identified as a key risk factor [175,252,263].

Pesticides such as paraquat and organochlorines are implicated in alveolar epithelial injury and fibrogenesis through oxidative stress and mitochondrial dysfunction [264,265]. Recent multi-omic data also highlight the role of environmental exposures in modulating key profibrotic mediators like TRIP13 [266]. These findings reinforce the need to address occupational and environmental risk factors in IPF prevention.

Epidemiological studies and case reports associate pesticide exposure, especially paraquat, organophosphates, and pyrethroids, with localized scleroderma and scleroderma-like conditions [267,268,269]. Paraquat, in particular, has been shown to inhibit collagen synthesis and induce oxidative stress and fibroblast dysfunction upon dermal contact [270,271,272]. Systemic effects following dermal pesticide absorption, including fibrosis-related outcomes, have been documented [273,274]. Emerging data also implicate immune dysregulation and epigenetic imprinting as central mechanisms by which occupational exposures promote fibrotic disease onset and progression [236,275]. Addressing these risks is essential to reducing the global burden of environmentally driven fibrosis.

## 6. Therapeutic Opportunities and Future Directions

Using a cross-organ lens, we have reviewed shared mechanisms of skin and lung fibrotic diseases involving fibroblast activation, immune dysregulation, ECM remodeling, and failure of resolution pathways. As clinical observations and molecular insights increasingly align, opportunities to repurpose initially approved antifibrotic therapies from the lung to the skin are rapidly expanding. Herein, we outline established and emerging therapies, including clinical trials that reflect this evolving landscape.

### 6.1. Evidence from SSc with Nintedanib

To date, evidence supporting the use of nintedanib in patients with SSc-ILD primarily stems from the SENSCIS trial and its post hoc analysis, and from subgroup data from the INBUILD trial. SENSCIS, a phase 3 randomized, double-blind, placebo-controlled trial, enrolled 576 patients across 32 countries and demonstrated that participants receiving nintedanib experienced a 44.5 mL smaller annual decline in FVC compared to the placebo group, indicating a beneficial effect on slowing disease progression. Combined use with mycophenolate mofetil (MMF) suggested a potential additive effect with acceptable tolerability, though improvement was restricted to pulmonary outcomes [276]. A post hoc analysis of the SENSCIS trial assessed changes in FVC% predicted using specific categorical thresholds, including 5%, 10%, and the established minimal clinically important differences for declines or increases in FVC, and reinforced the therapeutic relevance of nintedanib in altering disease trajectory [277]. Meanwhile, in the INBUILD phase 3 randomized controlled trial, which included 5.9% of participants diagnosed with SSc-ILD, nintedanib reduced the rate of FVC decline uniformly, regardless of underlying ILD diagnosis [278,279,280].

### 6.2. From the Lung to the Skin: Expanding Antifibrotic Therapies

Both pirfenidone and nintedanib, approved for IPF, inhibit key fibrotic signaling cascades, including TGF-β pathways, oxidative stress responses, and fibroblast proliferation. These agents attenuate pulmonary function decline and fibrotic tissue accumulation and are actively being evaluated in systemic sclerosis-associated interstitial lung disease (SSc-ILD). However, their efficacy in reversing established dermal fibrosis remains limited. In particular, in vitro studies have demonstrated that nintedanib can inhibit the proliferation, migration, and collagen production of dermal fibroblasts from SSc patients, suggesting a potential for antifibrotic effects on the skin [281]; yet in the SENSCIS trial, there was no significant difference in the change in mRSS between the nintedanib and placebo groups at week 52.

Nevertheless, mechanistic overlap between pulmonary and dermal fibrosis suggests potential benefit in chronic and refractory cutaneous fibrotic conditions such as longstanding scleroderma plaques, hypertrophic scars, and post-burn contractures where shared fibroinflammatory circuits are operative [95]. Notably, we have recently seen concurrent improvement in SSc patients with psoriatic plaques [282].

Similarly, mucosal Th2-driven diseases such as asthma exhibit structural remodeling including subepithelial fibrosis, goblet cell hyperplasia, and basement membrane thickening [283,284,285,286,287,288]. These fibrotic features parallel those observed in chronic atopic dermatitis (AD) and hidradenitis suppurativa (HS), positioning them as rational targets for the repurposing of antifibrotic therapeutics [289,290,291].

### 6.3. Fibroinflammatory Skin Diseases: A Paradigm Shift

Diseases such as HS and chronic AD are now recognized as fibroinflammatory with fibrotic changes. In HS, chronic lesions evolve into deep, fibrotic dermal tunnels characterized by TGF-β upregulation, α-SMA+ myofibroblasts, and elevated periostin expression [292,293,294]. Periostin is now targeted via antiperiostin antibodies, small molecule inhibitors, or RNA-based strategies in both IPF and cutaneous fibrosis [295,296,297]. Likewise, selective PDGFR inhibitors like crenolanib have shown efficacy in SSc fibroblasts [298]. Single-cell RNA sequencing has confirmed profibrotic fibroblast subsets in HS lesions [299]. In lichenified AD, IL-13–TGF-β crosstalk mediated fibroblast activation and thickening, challenging the sufficiency of anti-inflammatory monotherapy [289,291], underscoring the need for antifibrotic or combinatorial therapeutic strategies (Table 1).

Biologics such as dupilumab (IL-4Rα blocker) and lebrikizumab (IL-13 monoclonal antibody) target the IL-4/IL-13–TGF-β axis. They showed efficacy in ameliorating both inflammatory and early fibrotic changes in AD, asthma, and chronic rhinosinusitis with nasal polyposis (CRSwNP), and are under study in SSc and fibrosing pulmonary conditions [88,300,301,302,303,304,305,306,307,308,309,310,311,312,313,314,315,316,317,318]. Similarly, JAK inhibitors, such as baricitinib, tofacitinib, and ruxolitinib, may offer an additional route to disrupt fibrotic remodeling in SSc as in chronic AD [319,320,321,322,323,324,325,326]. Additionally, senolytic agents such as navitoclax (a Bcl-2 family inhibitor) and flavonoid fisetin clear senescent fibroblasts in both IPF and cutaneous fibrosis models [98,327,328,329]. Furthermore, B-cell depletion therapy (rituximab), T-cell–directed therapies (abatacept), and cell-based therapies (mesenchymal stem cells, MSCs, and CAR-T) are being evaluated as well, yet with limitations [330,331,332,333]. Cell-based therapies reduce autoimmunity, fibrosis, and allow immune reconstitution with a less auto-reactive repertoire [334,335,336].

### 6.4. RNA and Epigenetic Therapeutics

Shared fibrotic regulatory RNAs across the skin and lung offer translational opportunities for RNA-based therapies. The most promising are miR-29 mimetics (e.g., Remlarsen) and anti-miR-21 oligonucleotides, which modulate profibrotic gene networks and are in development for keloids, SSc, and IPF [162]. Epigenetic modulators, such as EZH2 and HDAC inhibitors (e.g., Trichostatin A), reprogram fibroblast transcriptional landscapes, while lncRNAs (e.g., FEZF1-AS1 and ANRIL) suppress upstream fibrotic circuits [139,147,151,152]. Innovative delivery systems, particularly engineered EVs, enable organ-specific RNA therapeutic delivery to fibrotic microenvironments, enhancing therapeutic precision while reducing off-target effects [337,338,339].

Despite their promise, epigenetic therapies face important challenges. HDACs and EZH2 show significant potential for treating fibrosis but face several obstacles. HDAC inhibitors often lack isoform specificity, leading to off-target effects such as the suppression of anti-inflammatory cytokines [129]. Although they are promising in animal models, their application in human clinical trials for conditions like IPF remains limited due to the complexity of fibrosis and incomplete understanding of HDAC isoform functions [340]. Conflicting data, especially regarding HDAC6, alongside the scarcity of comparative clinical studies in humans, demonstrates the need for deeper clinical and mechanistic research to assess efficacy and safety [341,342]. Regarding histone methyltransferase EZH2 inhibitors, although promising, they face some challenges, including low bioavailability, high molecular weight, and the need for high optimal doses and precise timing of administration, since inhibition at the wrong phase of inflammation can disrupt immune responses or tissue repair. The effects of EZH2 inhibition can also vary by cell type and specific disease context, reflecting the multifaceted nature of fibrosis, involving various signaling pathways (TGF-β, Notch, and Wnt/b-catenin) [343,344,345]. Early EZH2 inhibitors, such as DZNep, faced limitations due to their off-target effects against other methyltransferases. Some of these effects, such as reversible splenomegaly and temporary testis reduction, have been observed in animal models but appeared to be manageable [346]. However, recent advances offer improved specificity, longer-lasting pharmacodynamics, and minimal toxicity, even against many EZH2 mutations [347,348]. Future research should aim to develop selective inhibitors, clarify isoform-specific functions, and explore combination therapies to enhance effectiveness and minimize adverse effects.

Implementation of fibrosis-aware precision medicine in dermatology, especially for conditions like HS and AD, requires biomarker-driven patient stratification [349,350]. Candidate biomarkers such as periostin, miR-21, and miR-29 may predict patients at high risk for irreversible fibrotic remodeling and guide antifibrotic therapy selection [156,158,351].

Future directions include combinatorial clinical trials should evaluate the efficacy of topical or intralesional antifibrotics, RNA-based agents, and TGF-β inhibitors in patients with lichenified AD or tunnel-forming HS. In parallel, the repurposing of lung-targeted antifibrotics and immune biologics for fibrotic dermatologic diseases is an active area of investigation. Importantly, environmental exposure history, including pollutants, UV radiation, and thermal injury, may modulate fibrotic disease trajectory and should be systematically incorporated into precision therapeutic strategies.

## 7. Conclusions and Future Perspectives

Skin and lung fibrosis, though arising in distinct anatomical sites, are united by shared molecular, cellular, and environmental mechanisms. Persistent fibroblast activation, immune dysregulation, ECM remodeling, and the failure of resolution pathways constitute a common pathogenic axis that is amplified by environmental insults and epigenetic reprogramming. The convergence of these pathways not only deepens mechanistic insights but also paves the way for cross-organ therapeutic innovation.

The increasing influence of climate change, pollution, and occupational hazards on fibrotic diseases highlights the urgent need for a fibrosis-aware precision medicine approach. Emerging antifibrotic drugs repurposed from pulmonary disease, RNA- and epigenetic-based therapies, and biologics targeting IL-4/IL-13 pathways are demonstrating promising translational potential for skin fibrosis.

Looking ahead, therapeutic strategies must integrate the dynamic exposome and patient-specific molecular signatures. Clinical trials should integrate patient heterogeneity in drug efficacy, since retrospective analyses reveal differential treatment responses across subsets. Recognition and stratification of molecular subsets are critical for advancing precision medicine in SSc and related conditions. Beyond traditional immunosuppression, newer insights into understanding of fibrosis, vascular pathology, and immune mechanisms are guiding the development of targeted, mechanism-based interventions. Integrating environmental exposome data, molecular profiling, and organ-shared biomarkers (e.g., miR-21, periostin, lncRNAs) will enable stratified treatment approaches and improve outcomes. Multidisciplinary efforts bridging dermatology, pulmonology, immunology, and environmental health are essential for tackling the rising burden of fibrotic disease in an ever-changing world.

## Figures and Tables

**Table 1:** Emerging therapeutic strategies in skin and lung fibrosis. (**↑**: upregulation, **↓**: downregulation).

Strategy/Agent	Mechanistic Target	Fibrotic Pathway Modulation	Skin Relevance	Lung Relevance	Translational Notes
Pirfenidone	TGF-β, oxidative stress, fibroblast proliferation	Inhibits TGF-β signaling and ROS-mediated ECM accumulation	Investigated in SSc, keloids, hypertrophic scars	Approved for IPF, reduces FVC decline	SSc-ILD trials ongoing; dermal efficacy remains modest
Nintedanib	PDGFRα/β	Attenuates fibroblast activation and ECM synthesis	Effective in patient-derived fibroblasts from SSc	Targeting lung fibroblast heterogeneity in preclinical models	Cross-organ translational potential; periostin/PDGF crosstalk
Anti-IL-4/IL-13 Biologics (e.g., Dupilumab, Lebrikizumab)	IL-4Rα/JAK–STAT6 axis	Reduces type 2 inflammation, fibroblast activation, and periostin expression	Shown to improve lichenified AD and chronic HS remodeling	Approved for asthma/CRSwNP; reduces airway fibrosis	Trials in systemic fibrosis and lung-AD spectrum diseases
PDGFRiInhibitors (e.g., Crenolanib	PDGFRα/β	Attenuates fibroblast activation and ECM synthesis	Effective in patient-derived fibroblasts from SSc	Targeting lung fibroblast heterogeneity in preclinical models	Cross-organ translational potential; periostin/PDGF crosstalk
Periostin-targeted therapies	Periostin–integrin axis	Blocks fibroblast migration, ECM stiffening, and TGF-β feedback	HS, keloids, SSc plaques with high periostin expression	Elevated serum periostin in IPF; biomarker of progression	Validated biomarker and emerging therapeutic target
RNA-based Therapies (e.g., anti–miR-21, miR-29 mimics)	miRNAs (miR-21 ↑, miR-29 ↓)	Restores antifibrotic miRNA balance; targets TGF-β and ECM genes	Remlarsen (miR-29) for keloids/SSc; anti-miR-21 reduces dermal scarring	Anti-miR-21 attenuates IPF in vivo	Organ-shared regulatory RNAs enable dual indications
Epigenetic modulators (EZH2, HDAC inhibitors)	Chromatin modifiers, lncRNA–miRNA axis	Silences profibrotic transcription; reprograms fibroblast phenotype	HDAC inhibitors reduce collagen in keloid/SSc fibroblasts	EZH2 promotes EMT, ECM accumulation in IPF	Precision-targeted and cell-specific epigenetic therapies emerging
Senolytic Agents (e.g., Navitoclax, Fisetin)	Senescent fibroblasts, SASP	Induces apoptosis of fibrotic fibroblasts; reduces SASP cytokines	Reverses fibroblast persistence in keloid models; aging skin fibrosis	Preclinical efficacy in IPF and radiation-induced lung injury	Targets inflammaging and chronic remodeling loops
Extracellular Vesicle (EV) RNA Delivery	Organ-specific miRNA/lncRNA payloads	Delivers antifibrotic RNA cargo (e.g., miR-29 or miR-148a-3p) to fibroblasts	Experimental in dermal fibrosis, AD, burn injury	MSC-EVs mitigate lung fibrosis via Wnt, TGF-β suppression	Platform for precision, low-toxicity, cross-organ applications
JAK Inhibitors (e.g., Ruxolitinib)	JAK1/JAK2; downstream of IL-4/IL-13	Interrupts immune–fibrotic signaling (STAT6, IL-6, or TGF-β)	Efficacy in SSc, AD with fibrotic plaques	Investigational in fibrosing ILDs, SSc-ILD	Suitable for combined inflammatory–fibrotic phenotypes
